# Pre-existing humoral immunity to low pathogenic human coronaviruses exhibits limited cross-reactive antibodies response against SARS-CoV-2 in children

**DOI:** 10.3389/fimmu.2022.1042406

**Published:** 2022-10-19

**Authors:** Nina Li, XueYun Li, Jiani Wu, Shengze Zhang, Lin Zhu, Qiqi Chen, Ying Fan, Zhengyu Wu, Sidian Xie, Qi Chen, Ning Wang, Nan Wu, Chuming Luo, Yuelong Shu, Huanle Luo

**Affiliations:** ^1^ School of Public Health (Shenzhen), Shenzhen Campus of Sun Yat-sen University, Shenzhen, China; ^2^ School of Public Health (Shenzhen), Sun Yat-sen University, Guangzhou, China; ^3^ Shenzhen Institute of Advanced Technology, Chinese Academy of Sciences, Shenzhen, China; ^4^ Department of Epidemiology, Shenzhen Nanshan Center for Disease Control and Prevention, Shenzhen, China; ^5^ Institute of Pathogen Biology, Chinese Academy of Medical Sciences and Peking Union Medical College, Beijing, China; ^6^ Key Laboratory of Tropical Disease Control, Sun Yat-sen University, Ministry of Education, Guangzhou, China

**Keywords:** low pathogenic human coronaviruses, SARS-CoV-2, cross-reactive antibody, neutralizing activity, antigen specific antibody

## Abstract

Severe acute respiratory syndrome coronavirus 2 (SARS-CoV-2) infection causes asymptomatic or mild symptoms, even rare hospitalization in children. A major concern is whether the pre-existing antibodies induced by low pathogenic human coronaviruses (LPH-CoVs) in children can cross-react with SARS-CoV-2. To address this unresolved question, we analyzed the pre-existing spike (S)-specific immunoglobin (Ig) G antibodies against LPH-CoVs and the cross-reactive antibodies against SARS-CoV-2 in 658 serum samples collected from children prior to SARS-CoV-2 outbreak. We found that the seroprevalence of these four LPH-CoVs reached 75.84%, and about 24.64% of the seropositive samples had cross-reactive IgG antibodies against the nucleocapsid, S, and receptor binding domain antigens of SARS-CoV-2. Additionally, the re-infections with different LPH-CoVs occurred frequently in children and tended to increase the cross-reactive antibodies against SARS-CoV-2. From the forty-nine serum samples with cross-reactive anti-S IgG antibodies against SARS-CoV-2, we found that seven samples with a median age of 1.4 years old had detected neutralizing activity for the wild-type or mutant SARS-CoV-2 S pseudotypes. Interestingly, all of the seven samples contained anti-S IgG antibodies against HCoV-OC43. Together, these data suggest that children’s pre-existing antibodies to LPH-CoVs have limited cross-reactive neutralizing antibodies against SRAS-CoV-2.

## Introduction

Coronaviruses (CoVs) refer to a large family of viruses that cause illnesses ranging from the common cold to more severe diseases. There are seven identified coronaviruses causing human infections. The highly pathogenic human coronaviruses (HPH-CoVs) including severe acute respiratory syndrome coronavirus (SARS-CoV), middle east respiratory syndrome coronavirus (MERS-CoV), and SARS-CoV-2 belong to the betacoronaviruses, while two alphacoronaviruses (HCoV-229E, HCoV-NL63) and two betacoronaviruses (HCoV-HKU1, HCoV-OC43) are identified as low pathogenic human coronaviruses (LPH-CoVs). Prior to the huge morbidity and mortality caused by HPH-CoVs, the LPH-CoVs have long been circulating in humans and cause common cold with mild respiratory syndromes (1–5). The serological assays by detecting antibodies induced by LPH-CoVs are used to define the population’s herd immunity, and ≥90% of adults have antibody evidence against these four LPH-CoVs (6, 7). It is believed that the primary infection of LPH-CoVs commonly occurs in childhood, with repeated infection within 1-3 years and a higher infection frequency in children under 5 years old (8–11).

Though the LPH-CoVs induced antibody response is short-lasting and has limited protection from hosts infected by the same or different common cold coronaviruses (8), it is hypothesized that the cross-reactive antibodies response from prior LPH-CoVs exposure could have reduced the susceptibility and possibility of developing severe clinical syndrome on SARS-CoV-2 infection in children (12, 13). However, studies exploring whether the pre-existing antibodies induced by LPH-CoVs can cross-react with SARS-CoV-2 generate conflicting results. Some data showed that the pre-existing antibodies response in un-infected populations, especially in children and teenagers exhibited specific neutralizing activity against SARS-CoV-2 (14), and high levels of pre-existing immune responses against LPH-CoVs were associated with mitigating the disease severity of coronavirus disease 2019 (COVID-19) (15–17) or reduced the duration of symptom (18). Yet, other studies suggested a lack of SARS-CoV-2 cross-neutralization activity although antigen-specific antibodies response was detected from pre-pandemic serum samples of SARS-CoV-2 (11, 19, 20). These conclusions vary greatly in different cohorts which commonly include adults, and the cross-reactive antibodies against SRAS-CoV-2 in children with pre-existing LPH-CoVs humoral immunity need to be elucidated.

Here, we investigated the seroprevalence of LPH-CoVs in 658 serum samples obtained from hospitalized children prior to the SARS-CoV-2 pandemic and measured the cross-reactive antibodies against SARS-CoV-2. We observed that 40% to 60% of the serum samples contained spike (S)-specific immunoglobin (Ig) G antibodies for the different LPH-CoVs. Higher levels of the nucleocapsid (N)-, S-, and receptor binding domain (RBD)-specific IgG antibodies against SARS-CoV-2 were found in the LPH-CoVs exposed group, and re-infections with different LPH-CoVs appeared to increase the antigen-specific cross-reactive antibodies. However, limited neutralizing activity existed even for the samples with cross-reactive S-specific IgG antibodies against SARS-CoV-2.

## Materials methods

### Samples

A total of 658 pre-COVID-19 serum samples of children with respiratory infection symptoms (aged 0-15 years) collected between May 27 and December 15, 2019 were obtained from Guangzhou Women and Children’s Medical Center. 28 serum samples from SARS-CoV-2 patients with strongly neutralization activity against SARS-CoV-2 WT spike in micro-neutralization assay were obtained from Shenzhen Center for Disease Control and Prevention (21). All the experiments were performed in compliance with and under the approval of the biomedical research ethics committee, the public health school (Shenzhen) of Sun Yat-Sen University (2020–034).

### Plasmid and proteins

The env-deficient HIV-1 (pnl4-3.luc.R.-E-) plasmid expressing the luciferase reporter was constructed in our laboratory. The pcDNA3.1.SARS-CoV-2 WT spike plasmid was kindly provided by Dr. Yaoqing Chen of Sun Yat-sen University. cDNA of SARS-CoV-2 B.1.351(Delta) spike and SARS-CoV-2 P.1(Gamma) spike were synthesized and cloned into pcDNA3.1 vector. The proteins used in this study were purchased from Sino Biological (Beijing, China).

### Enzyme-linked immunosorbent assay

Antigen-specific IgG antibodies to SARS-CoV-2 N/S/RBD and HCoV-229E S/HCoV-NL63 S/HCoV-HKU1 S/HCoV-OC43 S were detected using a standard enzyme-linked immunosorbent assay (ELISA) (21). The optimal coating concentration of antigen and serum were 250 ng/well of SARS-CoV-2 N and S, 150 ng/well of SARS-CoV-2 RBD, 50 ng/well of HCoV-OC43 S, 30 ng/well of HCoV-229E S, HCoV-NL63 S and HCoV-HKU1 S and 1:500. A positive control (serum sample FS B26 with strongly neutralization activity in micro-neutralization assay is kindly provided by Guangdong Provincial Center for Disease Control and Prevention) diluted in ten-fold dilutions was set on every ELISA plate to normalize all the detected values. Besides, one serum from mice was used as a negative control on each plate to check the consistency of each plate.

### SARS-CoV-2 pseudovirus neutralization assay

HEK 293T cells were co-transfected with SARS-CoV-2 Spike plasmid and HIV-1 plasmid (pnl4-3.luc.R.-E-). Cell supernatant was collected at 48 hours post-transfection and virus titers were determined as described in detail previously (22). Serially diluted sera were added to a 96-well plate and incubated for 1 hour at 37°C. HEK 293T-ACE2 cells were added and plates were incubated for 48 hours. The luciferase activity level was assessed by Bright-Glo Luciferase Assay System (cat# E2620) purchased from Promega (USA), and 50% inhibitory concentration (IC_50_) was determined as the last serum dilution at which the titration curve matches inhibition equal to or above 50% of the 100% assay (21).

### Statistics

The finite mixture model was used to define the positive value of antibodies against the S antigen of LPH-CoVs. The individuals with a positive value of cross-reactive antibody against SARS-CoV-2 were defined by an OD value greater than the mean OD values minus two times the standard deviation of COVID-19 patients. The student’s *t* test was used to compare the differences between the two groups. A two-tailed *P* value<0.05 was considered statistically significant. The differences between male group and female group for each LPH-CoVs were compared by the χ2 test. Pearson’s correlation was used to test the association between the S-specific antibodies against LPH-CoVs with the cross-reactive N, S, and RBD antigen-specific antibodies against SARS-CoV-2. Statistical analysis of the clinical data was performed using SPSS Statistics version 25 software (IBM, Armonk, NY, USA). All the experimental data were analyzed in GraphPad Prism software (version 8) and R Studio software.

## Results

### Pre-existing antibodies response against LPH-CoVs in the children cohort

**Figure 1:**
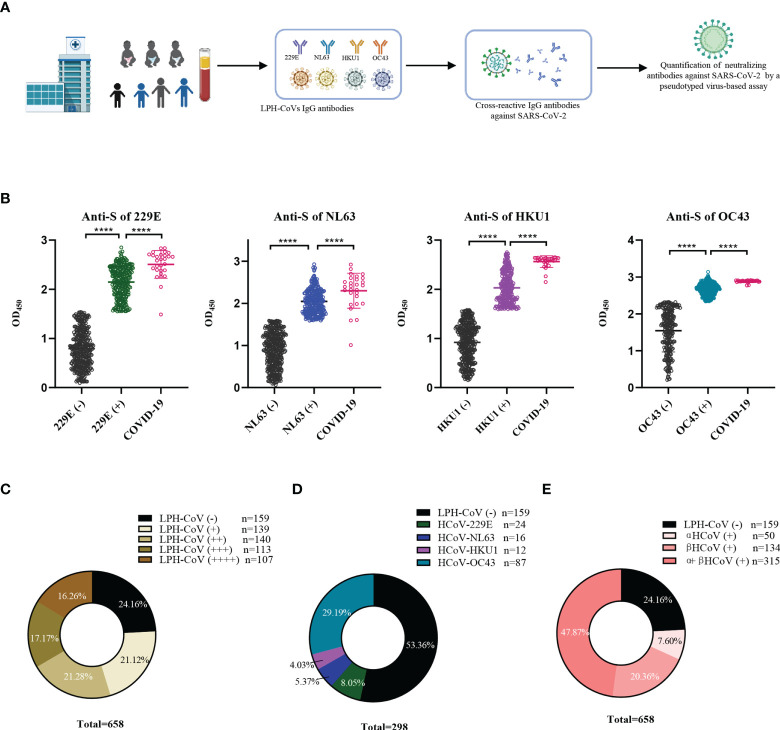
Identification of the pre-existing antibodies against LPH-CoVs in children’s serum samples **(A)** The schematic diagram of studying pre-existing antibodies against human coronavirus for the children cohort. **(B)** IgG antibodies response against the S protein of HCoV-229E, HCoV-NL63, HCoV-HKU1, and HCoV-OC43 was analyzed in 658 serum samples from hospitalized children prior to SARS-CoV-2 outbreak. The finite mixture model was used to define the individuals with positive values. 28 serum samples from convalescent COVID-19 patients were used as control. Student’s *t* test was used to compare the differences of medium values between groups, a two-tailed *P* value <0.05 was considered to be statistically significant, (****P ≤0.0001). **(C-E)** The percentage of individuals with exposure to different LPH-CoVs (n=658) **(C, E)** or only one of the four LPH-CoVs (n=298) **(D)**.

In this retrospective study, 658 serum samples collected from hospitalized children with a median age of 3 years (IQR 0.8-4.2) including 402 boys and 256 girls in 2019 before the SARS-CoV-2 outbreak were enrolled. The cohort’s pathological clinical features were summarized in **Supplementary Table 1**. To evaluate the pre-existing antibodies against LPH-CoVs in this cohort, we analyzed IgG antibodies response against the S antigen of HCoV-229E, HCoV-NL63, HCoV-HKU1, and HCoV-OC43 *via* ELISA (**Figure 1A**). Besides, 28 serum samples with neutralizing antibodies against SARS-CoV-2 from convalescent COVID-19 patients were used as an independent group. By utilizing the finite mixture model (**Supplementary Figures 1A-D**), we have observed that 40.73%, 38.30%, 39.67%, and 61.55% of the children’s serum samples were classified as positive individuals with antibodies against the S antigen of HCoV-229E, HCoV-NL63, HCoV-HKU1, and HCoV-OC43 respectively, suggesting a higher prevalence of HCoV-OC43 in hospitalized children than the other three LPH-CoVs in our cohort (**Figure 1B**). Interestingly, the COVID-19 patients boosted comparable S-specific IgG antibodies against these four LPH-CoVs (**Figure 1B**). Overall, 499 samples (75.84%) from the cohort exhibited S-specific IgG antibodies response against at least one of the four LPH-CoVs. Importantly, re-infections with different LPH-CoVs occurred commonly, we have found that 140/113/107 cases (21.28%, 17.17%, 16.26%) contained S-specific IgG antibodies against two/three/four of the LPH-CoVs respectively (**Figure 1C**). Among the 139 serum samples showing S-specific IgG antibodies only to one of the four LPH-CoVs, we observed positive rates of 8.05% for HCoV-229E (24 cases), 5.37% for HCoV-NL63 (16 cases), 4.03% for HCoV-HKU1 (12 cases), and 29.19% for HCoV-OC43 (87 cases) (**Figure 1D**). Besides, we have found a higher seroprevalence rate (20.36%) for beta LPH-CoVs compared to alpha LPH-CoVs (7.60%) in this cohort (**Figure 1E**). These results indicate that a high prevalence of LPH-CoVs exists in the early phase of human life, however, the pre-existing antibodies response is short-lasting to protect host from the same or other LPH-CoVs infection.

### Pre-existing antibodies response against LPH-CoVs in different age groups of the children cohort

**Figure 2:**
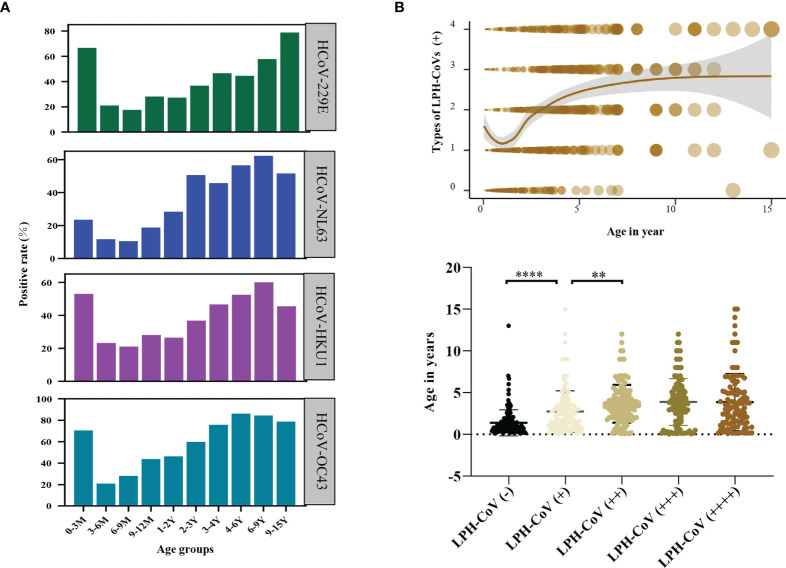
Analysis of the pre-existing antibodies response against LPH-CoVs in different age groups **(A)** The seropositive rates of HCoV-229E, HCoV-NL63, HCoV-HKU1, and HCoV-OC43 in different age groups. **(B)** The relation between age and re-infections of LPH-CoVs. The curve was fitted by Locally Weighted Regression (**B**, upper figure). A two-tailed *P* value <0.05 was considered to be statistically significant (**B**, lower figure), (**P values of ≤0.01, ****P ≤0.0001).

We next compared the seroprevalence in different age groups for positively classified serum samples with antibodies against LPH-CoVs. As shown in **Figure 2A**, we observed that the majority of infants less than 3 months old had S-specific IgG antibodies against HCoV-229E (66.67%), HCoV-HKU1 (52.94%), and HCoV-OC43 (70.59%), while 23.53% of the samples were seropositive individuals for HCoV-NL63, indicating a maternal transient S-specific IgG antibody in infants. Without maternal antibody, the numbers of seropositive individuals in the age category of 3 to 9 months old decreased sharply for all the four types of LPH-CoVs, then started to keep a whole upward trend following the age increase (**Figure 2A**; **Supplementary Figures 2A-D**). In the 9-15 years old group, we have found a reduced seropositive rate for HCoV-NL63, HCoV-OC43 and HCoV-HKU1, but a raised trend for HCoV-229E compared to the 6-9 years old group (**Figure 2A**). As expected, we have observed that re-infections of different coronaviruses increased with age under five years old and became stable afterward (**Figure 2B**).

**Figure 3:**
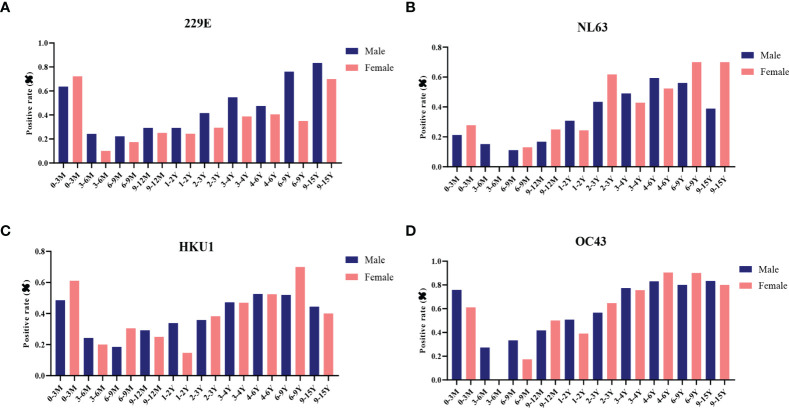
The effect of biological sex on pre-existing antibodies against LPH-CoVs in different age groups **(A-D)** The seropositive rates of HCoV-229E **(A)**, HCoV-NL63 **(B)**, HCoV-HKU1 **(C)** and HCoV-OC43 **(D)** in male and female individuals with different ages.

Next, the effect of biological sex on antibody levels of S-specific IgG against LPH-CoVs was analyzed. Due to the number difference of serum samples between boys and girls, we calculated the seropositive rates for boys and girls, respectively. Interestingly, boys had a higher positive rate compared with girls for HCoV-229E infection. However, no obvious gender-related pattern was found for the other three coronaviruses (**Figures 3A–D**; **Supplementary Table 2**).

### Identification of the cross-reactive antibodies against SARS-CoV-2 in the children cohort

**Figure 4:**
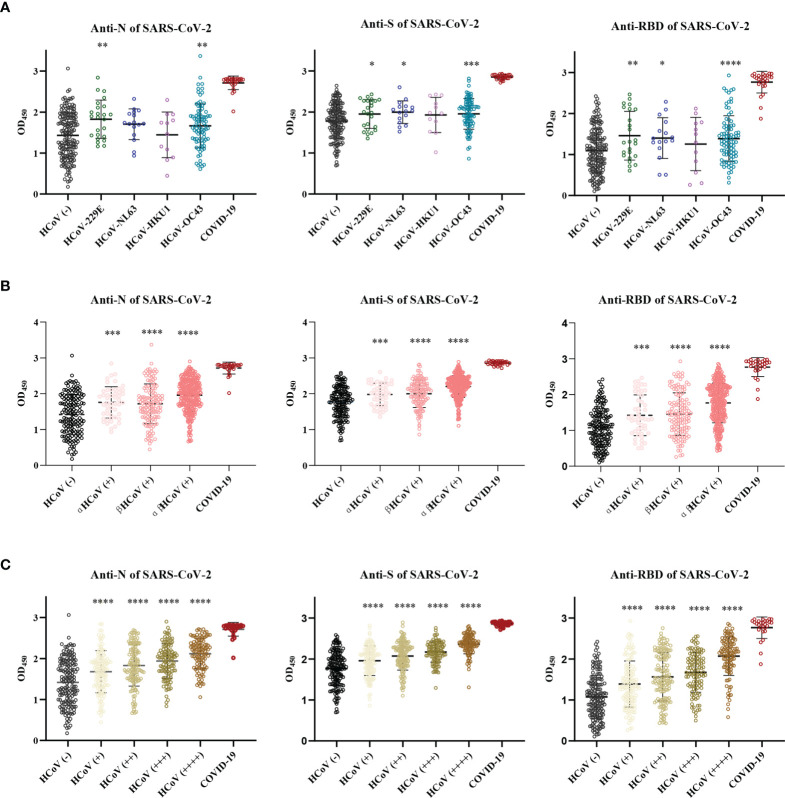
The pre-existing antibodies against LPH-CoVs contained cross-reactive antibodies against SARS-CoV-2 **(A-C)** The cross-reactive IgG antibodies against the N, S, and RBD proteins of SARS-CoV-2 in individuals with exposure to only one of the four LPH-CoVs, (n=298) **(A)** or re-exposed with different LPH-CoVs (n=658) **(B, C)**. The individuals with positive values were determined by an OD value greater than the mean OD values of COVID-19 patients minus two times the standard deviation of COVID-19 patients. A two-tailed *P* value<0.05 was considered statistically significant, (*P values of ≤ 0.05, **P values of ≤0.01, ***P values of ≤ 0.001, ****P ≤0.0001).

**Figure 5:**
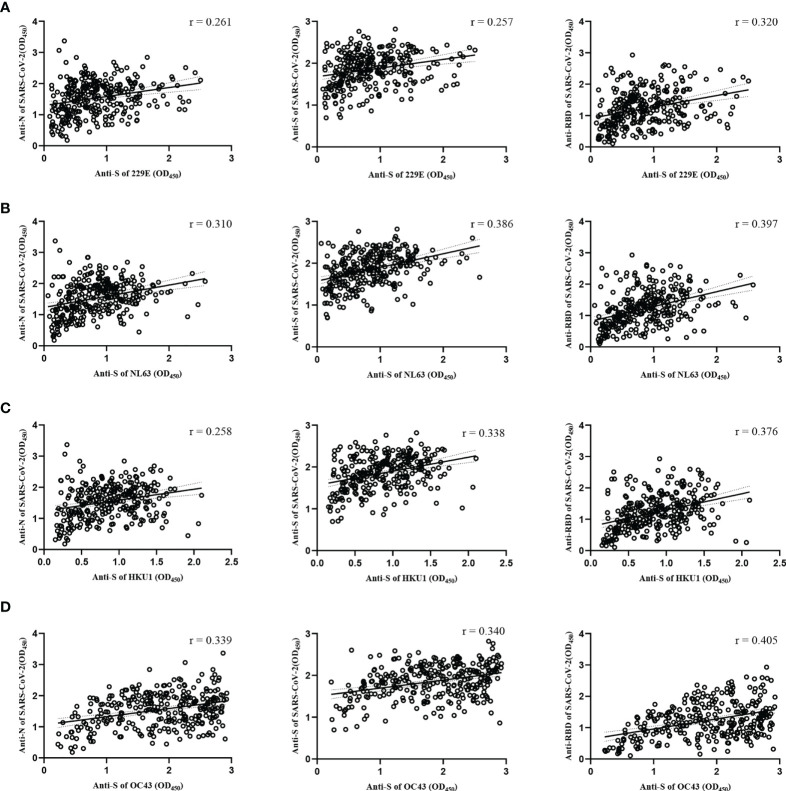
Correlations between the S-specific IgG antibodies against LPH-CoVs and the cross-reactive antigens-specific IgG antibodies against SARS-CoV-2 for the serum samples with only one of the LPH-CoVs exposure (n=298) **(A-D)** The correlations between the S-specific IgG antibodies against HCoV-229E **(A)**, HCoV-NL63 **(B)**, HCoV-HKU1 **(C)**, HCoV-OC43 **(D)** and the cross-reactive IgG antibodies against the N, S, RBD antigens of SARS-CoV-2 were assessed by Pearson correlation test. The *r* values were exhibited for the correlation. A two-tailed *P* value<0.05 was considered statistically significant.

To understand the cross-reactive antibody responses against SARS-CoV-2 induced by LPH-CoVs, we evaluated the IgG antibodies response against the N, S, and RBD antigens of SARS-CoV-2 in the serum samples using ELISA method. For the classified samples with only one of the four LPH-CoVs exposure, both HCoV-229E and HCoV-OC43 seropositive samples stimulated significantly higher levels of IgG antibodies response against the N, S, and RBD antigens of SARS-CoV-2 compared to the negative samples. The HCoV-NL63 seropositive samples contained elevated levels of IgG antibody response against the S and RBD proteins of SARS-CoV-2 rather than the N protein. Interestingly, no significant difference for the IgG antibodies response against the N, S, and RBD proteins of SARS-CoV-2 was found between the HCoV-HKU1 seropositive samples and the seronegative samples (**Figure 4A**). However, both alpha and beta coronaviruses seropositive samples did show enhanced IgG antibodies response against the N, S, and RBD proteins of SARS-CoV-2 compared with the negative samples (**Figure 4B**). As expected, we have found that the levels of IgG antibodies against the N, S, and RBD proteins of SARS-CoV-2 tended to increase with re-infections, and patients with all the four types of LPH-CoVs exposure had the highest levels of cross-reactive antigen-specific IgG antibodies against SARS-CoV-2 (**Figure 4C**). These results suggest that the repeated infections with LPH-CoVs may facilitate the host generating high levels of antigen-specific antibodies with cross-reactivity to SARS-CoV-2. We further determined the correlation between the S-specific IgG antibodies against LPH-CoVs and the cross-reactive antigen-specific IgG antibodies against SARS-CoV-2. For the serum samples with only one of the LPH-CoVs exposure, the positive correlations were noted between the cross-reactive antigens-specific IgG antibodies against SARS-CoV-2 with the S-specific IgG antibodies against each of the LPH-CoVs, especially with HCoV-OC43. (**Figures 5A–D**).

**Figure 6:**
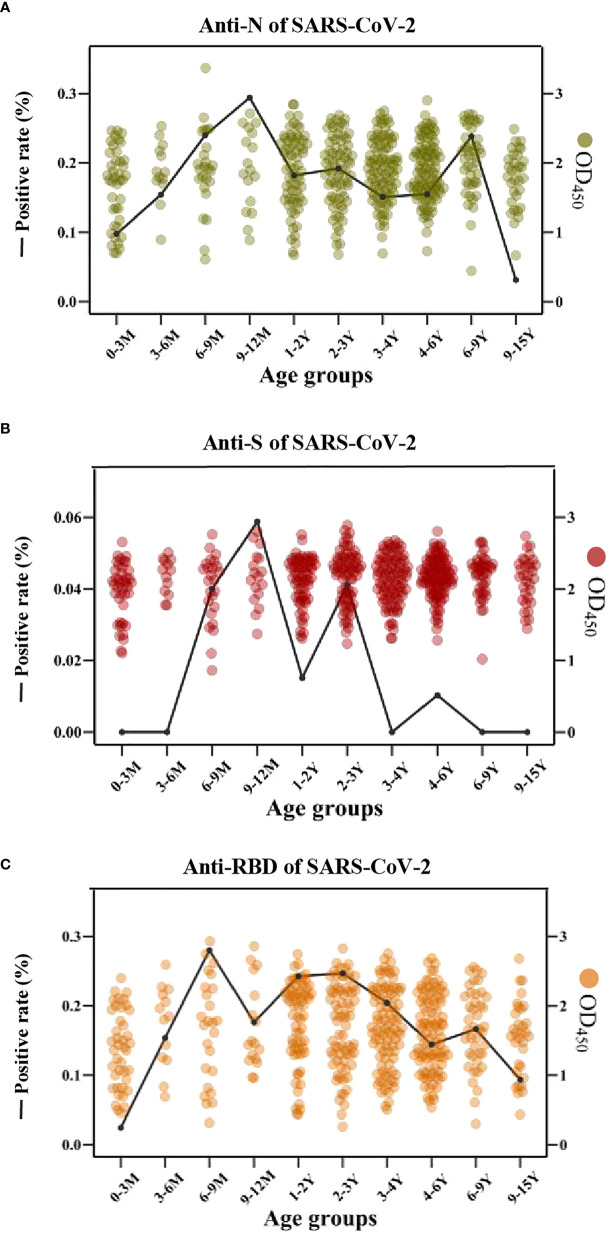
The cross-reactive IgG antibodies against SARS-CoV-2 in different age groups with pre-existing antibodies against LPH-CoVs **(A-C)** The prevalence (line) and OD values (dots) of cross-reactive IgG antibodies against the N **(A)**, S **(B)**, and RBD **(C)** of SARS-CoV-2 in different age groups.

Interestingly, the cross-reactive IgG antibodies against the N and S antigens of SARS-CoV-2 peaked mostly in the individuals with an age category of 9-12 months old, while the cross-reactive IgG antibody against the RBD antigen of SARS-CoV-2 peaked mostly in children of 6-9 months old (**Figures 6A–C**).

### LPH-CoVs stimulate limited cross-reactive neutralizing antibodies against SARS-CoV-2

**Figure 7:**
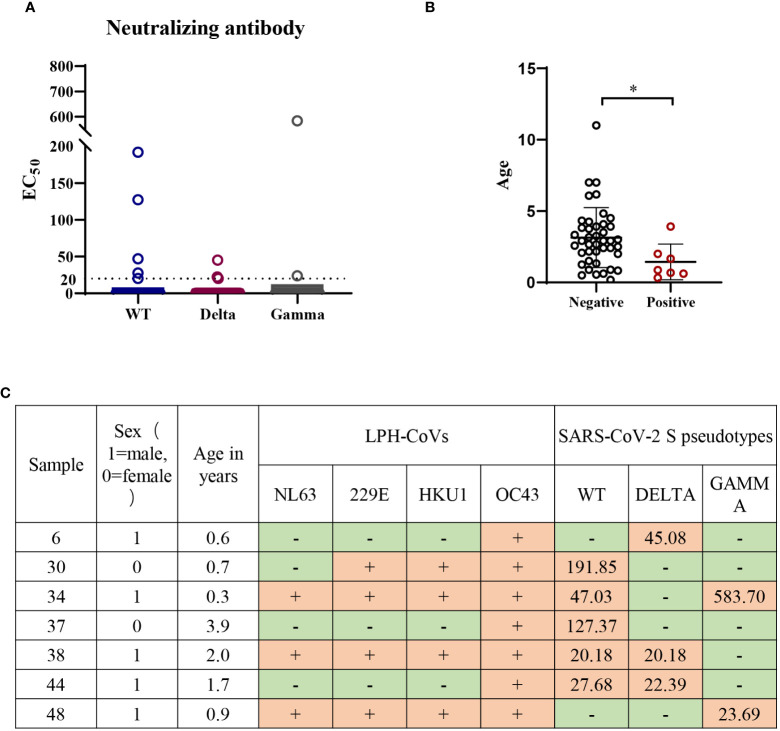
Low or undetectable neutralizing antibodies against WT or mutant SARS-CoV-2 S pseudotypes were found in the individuals with cross-reactive S-specific IgG antibodies against SARS-CoV-2 **(A)** The measurement of neutralization activity for the forty-nine serum samples with S-specific antibodies against SARS-CoV-2 *via* SARS-CoV-2 S pseudotypes neutralization assay. The dashed line represents a threshold set (EC_50_>20). **(B)** The age range for the serum samples with neutralizing antibodies against SARS-CoV-2 or variants. A two-tailed *P* value <0.05 was considered to be statistically significant, (*P values of ≤ 0.05). **(C)** The list of serum samples with neutralizing antibodies against SARS-CoV-2 S pseudotypes.

Forty-nine individuals with a positive cross-reactive S-specific IgG antibodies response against LPH-CoVs were determined by an OD value higher than two times the average of the COVID-19 patients. To examine whether the antibodies boosted by LPH-CoVs in these samples have neutralizing activity for SARS-CoV-2, we measured the neutralizing activity utilizing wild-type (WT) and two variants of concern (VOC) including Gamma and Delta SARS-CoV-2 S pseudotypes. As shown in **Figures 7A**, **C**, we have found that five serum samples could neutralize WT SARS-CoV-2 though the majority of selected samples lack neutralizing activity. Out of the five serum samples, two samples could neutralize Delta SARS-CoV-2 and one contained the neutralizing activity against Gamma SARS-CoV-2, indicating an immune escape of the variants. Interestingly, only one serum sample was found having the ability of neutralizing Delta or Gamma SARS-CoV-2 respectively, but without neutralizing activity against WT SARS-CoV-2. Notably, all of the serum samples with neutralizing activity against SARS-CoV-2 were collected from individuals under 4 years old, with of median age of median age of 1.4 years old (**Figures 7B, C** **)**, indicating the individuals with younger age may be beneficial to the cross-reactive neutralizing activity against SARS-CoV-2.

Next, we found all of the seven serum samples with cross-reactive neutralization activity against SARS-CoV-2 were HCoV-OC43 seropositive (**Figure 7C**), suggesting the pre-existing antibody against HCoV-OC43 may play a role in reducing SARS-CoV-2 infection.

## Discussion

The impact of pre-existing LPH-CoV-specific antibodies with cross-reactivity against SARS-CoV-2 has been investigated since the beginning of SARS-CoV-2 pandemic. It is critical if the antibodies boosted by LPH-CoVs can neutralize SARS-CoV-2 due to the 90% herd immunity of humans against LPH-CoVs (23, 24), which usually occur repeatedly in early life. In this study, we detected a generally moderate level of seroprevalence in 658 children under 15 years old for the three human coronaviruses (HCoV-229E, HCoV-NL63, HCoV-HKU1) and a high positive rate for HCoV-OC43. Although the seroprevalence of LPH-CoVs varies greatly by reason of different antigens used, methodologies adopted, population with distinct ages, and other demographic characteristics (25–27), the seroprevalence acquired from this cohort is somewhat in line with the normal range within children (28–30). Besides, the obvious maternal transient passive IgG antibodies response against LPH-CoVs was observed for infants within 3-months, which is consistent with the previous stratified studies (29, 31).

Limited studies have investigated whether biological sex affects LPH-CoVs infection although several reports have indicated that male COVID-19 patients appeared to be more susceptible to SARS-CoV-2 and generated a higher level of antibodies (21, 32). In this study, we found that HCoV-229E boosted higher S-specific IgG antibodies in boys than girls after 3 months old while no obvious sex-based antibody trend was observed for the other three LPH-CoVs. A recent study has found a higher level of IgG antibody against HCoV-229E other than the other three coronaviruses in men compared with women in an adult cohort (older than 20 years of age) (33). These data indicate that sex may be a factor affecting the prevalence of HCoV-229E.

Most adults commonly contain antibodies against all the four LPH-CoVs since re-infections occur initially in children, causing the difficulty of evaluating the cross-reactive antibody response to every specific coronavirus. In this study, we selected samples which are positive with only one type of IgG antibody against LPH-CoVs to investigate the cross-reactive antibody response against SARS-CoV-2, HCoV-HKU1 seropositive samples appeared to have no cross-reactive antibodies against the N, S, and RBD antigens of SARS-CoV-2. However, HCoV-OC43 seropositive samples showed significantly higher levels of, even comparable antigen-specific antibodies response against SARS-CoV-2 with the COVID-19 patients. Previous studies investigating whether the pre-existing anti-HCoV-OC43 antibodies affect SARS-CoV-2 infection yielded contradictory results. Several investigations have indicated high levels of N-specific antibodies against HCoV-OC43 in COVID-19 adult patients were associated with mild disease (34, 35), indicating a potential protective role of the pre-existing antibodies against HCoV-OC43 for SARS-CoV-2. On the other hand, Guo et al. have found significantly higher anti-S of HCoV-OC43 IgG antibody titers in patients with severe disease than those in mild patients, indicating the cross-reactive antibody may enhance the severity of COVID-19 patients (36). Unluckily, we were not able to track these patients in our cohort to assess the susceptibility or disease severity for SARS-CoV-2 infection. However, it is notable that all of the seven serum samples from children ≤ 4 years old with neutralizing activity against SARS-CoV-2 contained S-specific IgG against HCoV-OC43, indicating there may be a role for antibodies boosted by HCoV-OC43 in the earlier age during SARS-CoV-2 infection.

It is valuable that the serum samples of young individuals under 1-year-old were enrolled in this study. A prior study has reported that about 44% (21 cases in 48 samples in total) of the individuals had detectable S-specific IgG antibodies against SARS-CoV-2 in children ranging from 1-16 years *via* a flow cytometry-based method, most of which could neutralize SARS-CoV-2 (14). Another study has observed that 20% of SARS-CoV-2-free individuals contained antibodies against the N, S RBD antigens of SARS-CoV-2, but with very low or undetectable neutralizing antibodies in a cohort ranging from 1-90 years. Besides, they also found age did not affect the cross-reactive antibodies (11). Consistent with this report, we have found about 24.64% of individuals with pre-existing LPH-CoVs antibodies contained the cross-reactive antigen-specific antibodies against SARS-CoV-2, most of which contained undetectable neutralizing antibodies. However, all the seven samples with neutralizing activity against SARS-CoV-2 were collected from children under 4 years old in our cohort. Notably, four samples were acquired from infants born within 1 year old in a total of eleven samples with positive S-specific antibodies against SARS-CoV-2 in this age group, indicating the cross-reactive antibodies against SARS-CoV-2 may be higher in the earlier life with LPH-CoVs exposure. Consistently, several studies have reported that the pre-existing antibodies boosted at a very early life can bind the S protein of SARS-CoV-2, which might be an explanation for mild or no symptoms following SARS-CoV-2 in children (14, 37, 38). Interestingly, the re-infections with age did not influence the neutralizing activity although it appeared to increase the cross-reactivity antigen-specific antibodies levels. Nevertheless, other studies have found that vaccination of measles-mumps-rubella or tetanus-diphtheria-pertussis provided a protective role against the severe COVID-19 by activating the cross-reactive T cell response, which is a possible explanation for children with reduced susceptibility and severe clinical syndrome in SARS-CoV-2 infection (39–41). Thus, the cross-reactive T cell response should be an important point for future study since poor cross-reactive neutralizing antibodies are stimulated by LPH-CoVs. Due to the lack of COVID-19 children, the SARS-CoV-2 positive samples used here as control were collected from adult COVID-19 patients. Although comparable S-specific IgG antibodies against all the four LPH-CoVs were boosted in SARS-CoV-2 patients, we could not get a solid conclusion about the memory antibodies since the baseline of antibodies against LPH-CoVs has not been measured before they were infected with SARS-CoV-2.

Overall, our data demonstrate that the pre-existing antibody response boosted by LPH-CoVs has a moderate to high seroprevalence in children under 15 years old. However, the majority of pre-existing antibodies lack the neutralizing activity against SARS-CoV-2. Besides, HCoV-OC43 has a higher prevalence and may boost the cross-reactive neutralizing antibody in children under four years old against SARS-CoV-2, providing an insight into immunogen design and vaccine development.

## Data availability statement

The original contributions presented in the study are included in the article/**Supplementary Material**. Further inquiries can be directed to the corresponding authors.

## Ethics statement

All the experiments were performed in compliance with and under the approval of the biomedical research ethics committee, the public health school (Shenzhen) of Sun Yat-Sen University (2020-034).

## Author contributions

HL and YS conceived and designed the study. NL and XL performed experiments, analyzed data, and wrote the manuscript. JW, SZ, and LZ assisted in laboratory experiments. LZ and QiqC contributed to the data collection, formal analysis, and data interpretation. YF and SX collected clinical samples and data. QiC and NiW prepared experimental review materials. ZW, NaW, and CL provided experimental technical guidance. HL and YS approved the final version of the manuscript. All authors contributed to the article and approved the submitted version.

## Funding

This work was supported by the National Key Research and Development Program of China (2021YFC2300100, 2021YFC086300), Shenzhen Science and Technology Program (KQTD20200820145822023, KQTD20180411143323605), National Natural Science Foundation of China (32000116, 32270147), Shenzhen Science and Technology Program (JSGG20200225152008136, GXWD20201231165807008, and 20200825113322001, JCYJ20190807155407443, KCXFZ20211020172545006), High Level Project of Medicine in Nanshan, Shenzhen; Sanming Project of Medicine in Shenzhen (SZSM202103008).

## Acknowledgments

We would like to thank Director Bing Zhu and fellows in the Central laboratory of Guangzhou Women and Children’s Medical Center for their support in the collection of children samples in this study.

## Conflict of interest

The authors declare that the research was conducted in the absence of any commercial or financial relationships that could be construed as a potential conflict of interest.

## Publisher’s note

All claims expressed in this article are solely those of the authors and do not necessarily represent those of their affiliated organizations, or those of the publisher, the editors and the reviewers. Any product that may be evaluated in this article, or claim that may be made by its manufacturer, is not guaranteed or endorsed by the publisher.
