# Supplementary material for: Pre-existing humoral immunity to low pathogenic human coronaviruses exhibits limited cross-reactive antibodies response against SARS-CoV-2 in children
Source: Front Immunol. 2022 Oct 19;13:1042406. doi: 10.3389/fimmu.2022.1042406 (PMC9626651; doi:10.3389/fimmu.2022.1042406)
Supplement: Supplementary file 1 [file Table_1.docx]

**Supplementary Table 1.** Demographic characteristics of the study population

|  | **N** | **%** |
| --- | --- | --- |
| Cases | 658 | \ |
| Age (years) Median (IQR)* | 3.0 | 0.8-4.2 |
| Gender,n (%) |  |  |
| Male | 402 | 61.00 |
| Female | 256 | 38.85 |
| Symproms,n (%) |  |  |
| Cough | 218 | 33.08 |
| Fever | 199 | 30.20 |
| Gastrointestinal Symptoms (diarrhoea, abdominal pain, vomiting） | 50 | 7.59 |
| Convulsion | 24 | 3.64 |
| Twitch | 14 | 2.12 |
| Dizziness and headache | 6 | 0.91 |
| Intracranial infection/encephalopathy | 16 | 2.43 |
| Respiratory diseases（%） |  |  |
| Respiratory tract infections | 30 | 4.55 |
| Nasal, tonsils, laryngitis/inflammation of the upper respiratory tract | 35 | 5.31 |
| Bronchitis | 129 | 19.58 |
| Bronchopneumonia | 178 | 27.01 |
| Community-acquired pneumonia | 81 | 12.29 |
| Other pneumonia | 51 | 7.74 |

**Supplementary Table 2.** The number of cases with LPH-CoVs exposure in female and male individuals

|  | **Sex** | **Negative** | **Positive** | **Rate（%）** | ***P* value** |
| --- | --- | --- | --- | --- | --- |
| **229E** | Male | 224 | 178 | 44.28 | *0.020 ** |
|  | Female | 166 | 90 | 35.16 |  |
| **NL63** | Male | 255 | 147 | 36.57 | *0.252* |
|  | Female | 151 | 105 | 41.02 |  |
| **HKU1** | Male | 245 | 157 | 39.05 | *0.688* |
|  | Female | 152 | 104 | 40.63 |  |
| **OC43** | Male | 155 | 247 | 61.44 | *0.943* |
|  | Female | 98 | 158 | 61.72 |  |

The seropositive rates for LPH-CoVs were calculated in female and male individuals. The differences between the two groups for each LPH-CoVs were compared by the χ2 test. A two-tailed *P* value <0.05 was considered to be statistically significant.
