# Supplementary material for: Pre-existing humoral immunity to low pathogenic human coronaviruses exhibits limited cross-reactive antibodies response against SARS-CoV-2 in children
Source: Front Immunol. 2022 Oct 19;13:1042406. doi: 10.3389/fimmu.2022.1042406 (PMC9626651; doi:10.3389/fimmu.2022.1042406)
Supplement: Supplementary file 2 [file DataSheet_1.pdf]

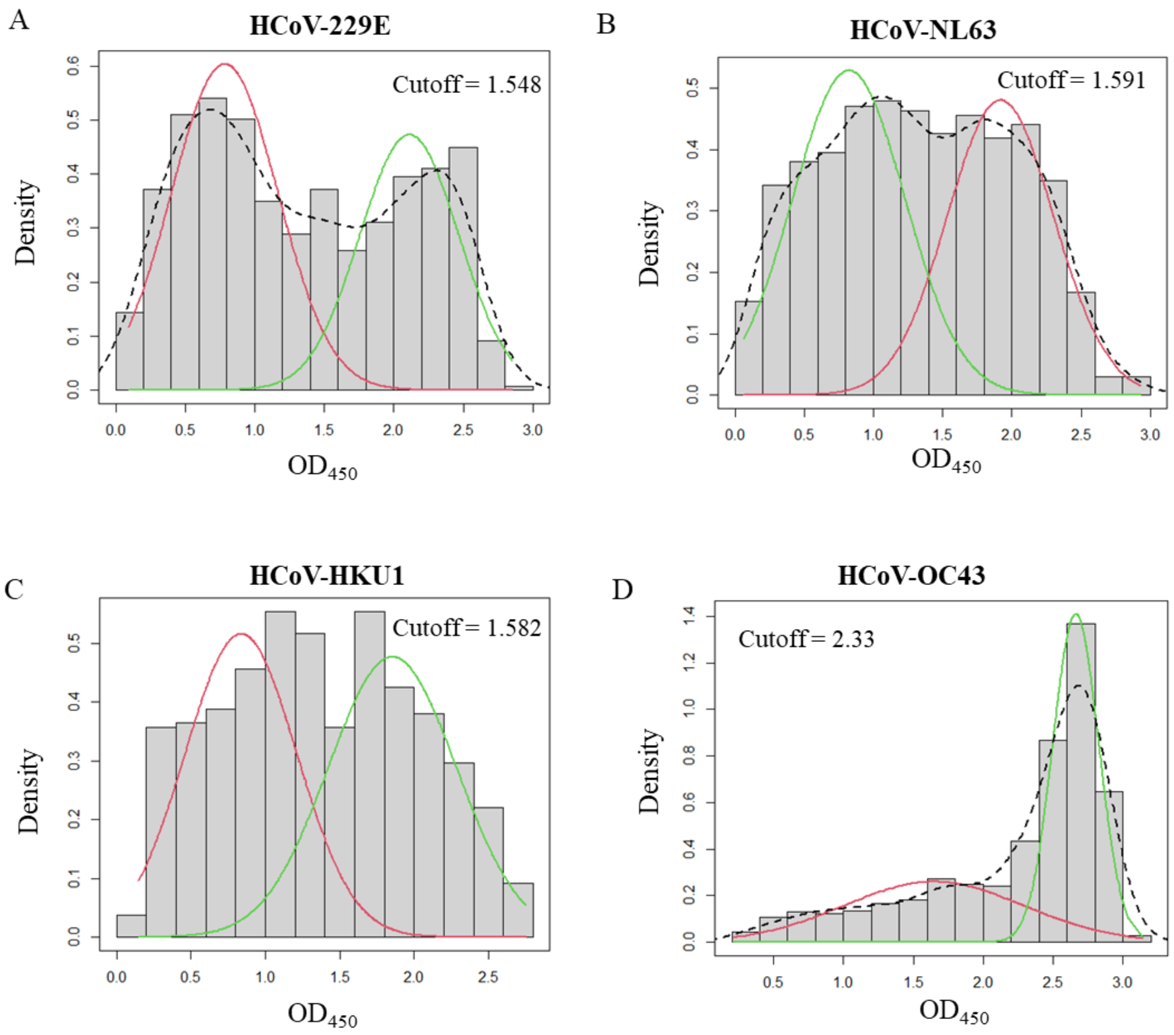

Supplementary Figure 1. Distribution of OD values for LPH-CoVs

(A-D) The density histogram of OD values for HCoV-229E (A), HCoV-NL63 (B), HCoV-HKU1 (C), and HCoV-OC43 (D). The finite mixture model plot was used for the cutoff value of positive serum samples. The cut-off value was the mean OD values of the seronegative population plus two times the standard deviation of the seronegative population (A-C), or the mean OD values of the seropositive population minus two times of standard deviation of the seropositive population (D).

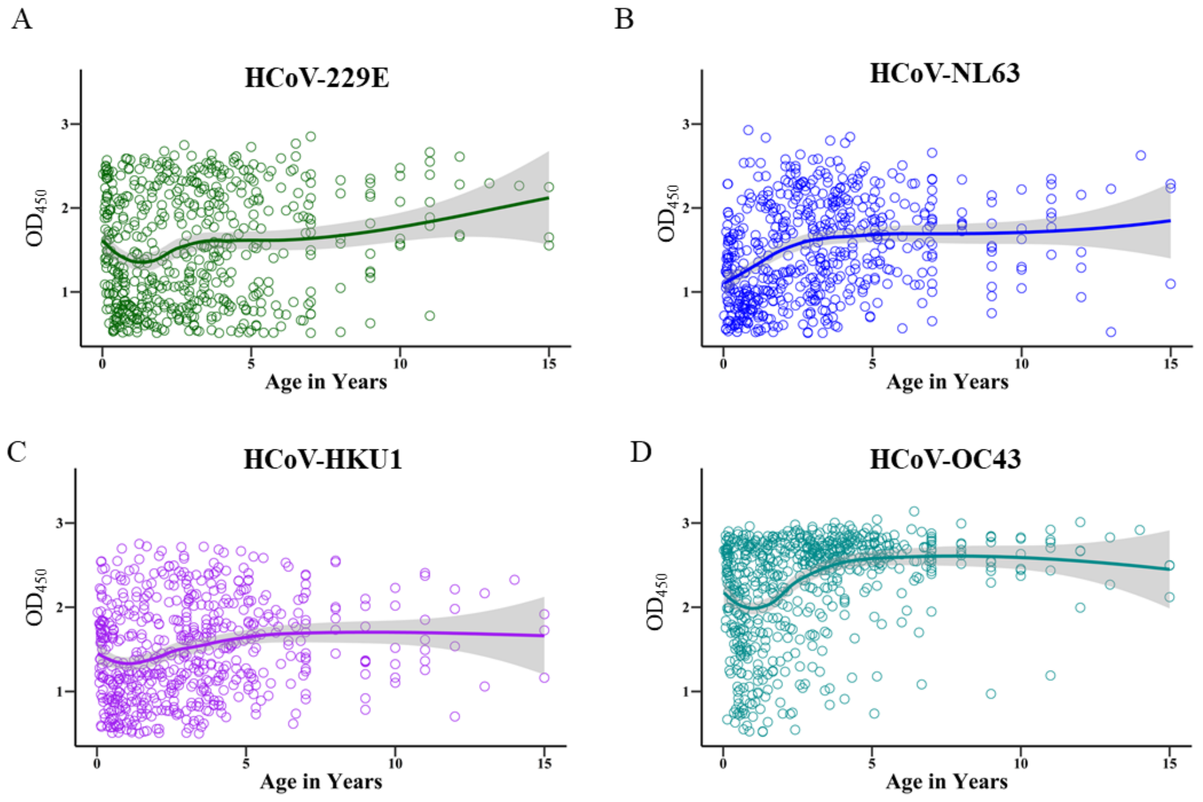

Supplementary Figure 2. The S-specific IgG antibodies against LPH-CoVs in different age groups

(A-D) Association between the S-specific IgG antibodies against HCoV-229E (A), HCoV-NL63 (B), HCoV-HKU1 (C), and HCoV-OC43 (D) and age. The curve was fitted by Locally Weighted Regression.
